# Vancomycin variable Enterococci in the Netherlands (2018–2023) and the mechanism of resistance induction

**DOI:** 10.1371/journal.pone.0342092

**Published:** 2026-02-06

**Authors:** Christian J. H. von Wintersdorff, Milika Roelofsen, Linda Versteegh, Yassin Benyahya, Casper Jamin, Marlies Mulder, Guido J. H. Bastiaens, Maurits P. A. van Meer, Jacky Flipse

**Affiliations:** 1 Department of Medical Microbiology and Infection prevention, Maastricht University Medical Center+, Maastricht, the Netherlands; 2 Department of Medical Microbiology and Immunology, Rijnstate Hospital, Arnhem, the Netherlands; Leibniz Institute DSMZ-German Collection of Microorganisms and Cell Cultures GmbH: Leibniz-Institut DSMZ-Deutsche Sammlung von Mikroorganismen und Zellkulturen GmbH, GERMANY

## Abstract

Enterococci are common human commensals but can cause severe infections in immunocompromised patients. *Enterococcus faecium* is a notable example, capable of acquiring resistance to multiple antibiotics, including the critically important drug vancomycin. Such strains, known as vancomycin-resistant *E. faecium* (VRE), are routinely detected in clinical laboratories using phenotypic assays. However, some isolates carry vancomycin resistance genes yet remain phenotypically susceptible; these are termed vancomycin variable enterococci (VVE). Because phenotypic assays may fail to identify VVE, patients treated with glycopeptides risk developing undetected VRE infections. VVE have been reported in Scandinavia and Japan, but their prevalence in the Netherlands remains largely unknown. To address this gap, two large Dutch clinical microbiology laboratories collaborated to screen enterococcal isolates for vancomycin resistance genes using molecular assays. Among 477 isolates tested, six carried *van* genes while remaining vancomycin-susceptible. Three of these subsequently developed vancomycin resistance *in vitro*. All three were *Enterococcus faecium* ST117 strains carrying a chromosomal *vanB2* operon, likely linked to the same outbreak. Genomic analysis revealed three mutations in the van operon regulator proteins: vanR (T189K) and vanS (G253C, L282V). We conclude that: (1) VVE are present in the Dutch population and may spread between patients; (2) VVE can develop into VRE upon vancomycin exposure; (3) specific mutations in regulatory proteins may underlie this phenotype; and (4) diagnostic policies should balance the low prevalence of VVE against their potential to cause severe complications, using sensitive molecular tests when appropriate. Our findings emphasize the importance of surveillance in revealing hidden threats and guiding clinical microbiology strategies, particularly with respect to VVE as precursors of VRE.

## Introduction

Enterococci are Gram-positive cocci that normally colonize the healthy human gut, with *Enterococcus faecalis* and *Enterococcus faecium* being the most common species. In immunocompromised individuals, however, enterococci can cause serious infections such as urinary tract infections, bacteraemia, and endocarditis [1]. These infections are typically treated with amoxicillin or vancomycin, but *E. faecium* often is resistant to amoxicillin, making vancomycin critically important in clinical practice [2]. As a result, vancomycin-resistant *Enterococcus faecium* (VRE) ranks ninth on the WHO’s list of Bacterial Priority Pathogens and is recognized as one of the ESKAPE pathogens (*E. faecium*, *S. aureus*, *K. pneumoniae*, *A. baumannii*, *P. aeruginosa*, and *Enterobacter* spp.) [3–5]. These pathogens are notorious for their antibiotic resistance and their central role in hospital-acquired infections [3,5].

In vancomycin-susceptible enterococci (VSE), vancomycin inhibits cell wall synthesis by binding to the D-Ala-D-Ala residues of pentapeptides, thereby blocking cross-linking of the mature cell wall. Resistance in VRE arises when these residues are replaced by D-Ala-D-Lac (resulting in a 1000-fold reduction in binding affinity) or D-Ala-D-Ser (3- to 8-fold reduction) [6–10]. High-level resistance is typically conferred by *vanA, vanB, vanD,* or *vanM* (D-Ala-D-Lac), while low-level of resistance is associated with *vanC, vanE, vanG, vanL,* or *vanN* (D-Ala-D-Ser) [6–10]. Characteristic vancomycin resistance types and MIC ranges include *vanA* (>128mg/L)*, vanB* (16–64 mg/L)*, vanC* (2–32 mg/L)*,* and *vanD* (8 to >128mg/L) [6–9]. Expression of these resistance genes is regulated by the two component regulatory system composed of the glycopeptide sensor (vanS) and transcriptional regulator (vanR) [11,12]. In the presence of vancomycin, vanS phosphorylates vanR, which then activates transcription of the *van* gene cluster.

Notably, some enterococci carry vancomycin resistance genes while remaining phenotypically susceptible. These so-called vancomycin-variable enterococci (VVE) pose a significant diagnostic challenge, as they are not detected by standard culture-based methods [13,14]. Clinically, this is of concern because VVE can convert from a susceptible to a resistant phenotype during vancomycin therapy [14], and – like VRE – can spread within healthcare institutions [11,15]. The emergence of *vanA*-positive VVE, including ST1421-CT1134 and ST203, in Denmark and Norway illustrates this risk [15,16]. Consequently, it has been recommended that invasive, phenotypically susceptible enterococci be screened for *vanA* using molecular methods [15], as phenotypic testing alone cannot detect VVE [16].

In the Netherlands, little is known about the prevalence of VVE, as national diagnostic guidelines rely exclusively on phenotypical assays. To address this gap, two large clinical microbiology laboratories in the southeastern region of the country collaborated to investigate the presence of VVE using molecular methods. In addition, VVE isolates identified in this study were further examined to determine their potential to shift from a susceptible to a resistant phenotype under vancomycin exposure.

## Materials and methods

### Selection and screening of Enterococcal isolates

Enterococcal isolates were collected without targeting specific patient populations and included both recent and archived strains. Isolates were retrieved from routine cultures and from long-term storage freezers (−70 °C) at Maastricht UMC+ (Maastricht, the Netherlands) and Rijnstate Hospital (Arnhem, the Netherlands), covering the period 2018–2023. The Maastricht UMC+ collection primarily included phenotypically vancomycin-susceptible isolates, whereas the Rijnstate collection comprised all enterococci cultured during the study period as well all isolates stored long-term, most of which originated from sterile sites or deep infections (e.g., periprosthetic tissue biopsies, ascites, pus).

Bacterial identification was performed using the Maldi Biotyper SMART system (Bruker Daltonik GmbH, Germany) at Rijnstate and the VITEK® MS (bioMérieux, Marcy-l’Étoile, France) at Maastricht UMC + . In total, 479 isolates were included: 228 (48%) from Maastricht UMC+ and 251 (52%) from Rijnstate Hospital. Isolates were cultured on blood agar plates with 5% sheep blood (Thermo Scientific Oxoid™, PB5012A, Rijnstate; BD, Franklin Lakes, New Jersey, SKU#221263, Maastricht).

Antimicrobial susceptibility testing was performed according to EUCAST guidelines (v14.0) after 24h incubation [17]. An 0.5 McFarland suspension was plated on Mueller-Hinton agar (Thermo Scientific Oxoid™, D10376) and overlaid with a vancomycin disk (5 μg, Liofilchem, 9164). After overnight incubation at 37 °C, the zone of inhibition was measured. Vancomycin resistance was defined as MIC > 4 mg/L (E-test, BioMérieux, France; or MIC Test Strip, Liofilchem, Italy) or a disk diffusion zone <12 mm (5 μg disk, Liofilchem, Italy). Amoxicillin resistance was defined as MIC > 8 mg/L (Vitek, BioMerieux, France; or E-test, BioMérieux, France or MIC Test Strip Liofilchem, Italy). Teicoplanin resistance was defined as a disk diffusion zone <16 mm (30 μg disk, Liofilchem, France).

### Molecular screening for vancomycin resistance genes

At Rijnstate, DNA was prepared by mixing 50 μL of bacterial suspension (1:10,000 dilution of a 0.5 McFarland suspension) with 100 μL achromopeptidase (Sigma-Aldrich, #065788, 1U/ μL) and 20 μL of internal control (phocid herpes virus type 1; European Virus Archive). After 15s vortexing, the mixture was incubated at 37 °C for 15 minutes, then at 95 °C for 5 minutes, followed by centrifugation (>10,000g for 1 min). Ten microliters of supernatant was used for PCR. PCRs targeting *E. faecium*-specific *recG, vanA, vanB, vanC* and *vanD* were performed as described in S1 Table [8,18]. PCRs were run on an ABI7500 FAST (Applied BioSystems, Forster City, CA, USA), using Fast Advance MasterMix with a cycling protocol of 2 minutes at 50 °C, 20 seconds at 95 °C, followed by 45 cycles of 95 °C for 3 seconds and 60 °C for 30 seconds.

At Maastricht, single colonies were inoculated into 350 µl TSB broth (Xebios Diagnostics GmbH, Düsseldorf, Germany). Nucleic acids were extracted from 280 µl broth plus 20 µl internal control (mCMV-gb DNA [8]) using the chemagic™ Viral DNA/RNA 300 Kit H96 on a a Chemagic 360D system (Revvity) with a Janus liquid handler. Extracts were eluted in 100 µl buffer, and 8 µl was used per PCR reaction. Reactions were performed in 20 μL total volume with TaqPath™ BactoPure™ Microbial Detection Master Mix (Thermo Scientific) on a Quantstudio 5 Real-Time PCR System (Applied Biosystems, Foster City, CA, USA). Cycling consisted of 2 minutes at 95 °C, followed by 42 cycles of 95 °C for 10 seconds and 60 °C for 30 seconds.

At both sites, Ct values <30 were considered positive. Higher values were retested. Positive and internal controls were assessed according to laboratory-specific protocols, and values should be within two standard deviations of the mean Ct value [19].

### Induction of resistance

Enterococci positive for any *van* gene, yet phenotypically susceptible for vancomycin were selected for induction of resistance. To this end, an 0.5 McFarland suspension was plated on a Mueller-Hinton agar (Thermo Scientific Oxoid™, D10376) and overlaid with a vancomycin disk (5 μg, Liofilchem, 9164). After overnight incubation at 37 °C, the zone of inhibition was measured. Thereafter, bacteria growing just outside the zone of inhibition were harvested and the process was repeated as described above. The process was repeated at least four times (when no resistance was induced) or six times (when resistance was induced and maintained). Both the first isolate (‘Susceptible’) and the last isolate (‘Resistant’) are selected and stored at −70 °C for further investigations.

### Sequencing and bioinformatic analysis

Whole genome sequencing (WGS) was performed as previously described [8]. Briefly, DNA was extracted using Lucigen MasterPure total DNA and RNA extraction kit (Lucigen, Middleston, WI, USA). Short read sequencing was done using Illumina MiSeq V2 2x250 cycles with NexteraXT library preparation kit according to manufacturer’s protocol. Long read sequencing libraries were made using the Rapid barcoding kit (SQK-RBK114.96). Sequencing was performed on a R10.4.1 flowcell and ran for 72 hours. Minimal sequencing depth for both short and long read sequencing was aimed at least 30x coverage. *De novo* hybrid assemblies were made using unicycler (v0.5.0). AMR and virulence gene detection was done using ABRicate (v1.0.1) with NCBI database and virulencefinder database [20]. SNP analyses was done using SKA on raw Illumina reads instead of assemblies [21], as this mitigates more sequencing and assembly errors [22]. All bioinformatic tools were run with default parameters unless otherwise specified. All sequencing reads were deposited in SRA, and are available under BioProject PRJNA1186210.

### RNA analysis

An 1μL inoculation loop full of bacterial growth was harvested from designated area’s on the agar plate and resuspended in 200 μL RNAse free water with 2 μL RNAse inhibitor (New England Biolabs, Ipswich, MA, USA, #M0314L) for a final concentration of 0.4 U/ μL). Subsequently, total nucleic acids were liberated by boiling the suspension at 95 °C for 5 minutes, followed by cooling on ice. The suspension was split in two portions of 100 μL each, one portion was treated with 1 U DNAse-I (Thermo Scientific, Waltham, MA, USA, #15198325) and left to incubate at 37 °C for 5 minutes, followed by inactivation of DNAse-I at 75 °C for 5 minutes. The resulting suspensions were centrifuged at full speed for 5 minutes. Clarified supernatant was used in the PCR. The Rijnstate PCR reaction (*vanA, vanB, recG*), described above, was used however replacing Fast Advance Mastermix 2x with Fast Viral Mastermix 4x (Thermo Scientific, Waltham, MA, USA). Analysis was conducted on the QuantStudio 5 (Thermo Scientific, Waltham, MA, USA), with a thermal cycling consisting of 5 minutes at 50 °C, 20 seconds at 95 °C followed by 45 cycles of 15 seconds at 95 °C and 1 minute at 60 °C. Results were analyzed per bacterial isolate cultured in the presence or absence of vancomycin according to the ΔΔCt method [23] by normalizing the *vanB* relative to the *recG* using the formula: ΔΔCt = (Ct_vanB_ - Ct_recG_)_far away_ – (Ct_vanB_ - Ct_recG_)_near_. Fold change of vanB expression due to proximity to vancomycin disk is calculated as 2^(-ΔΔCT).

### Vancomycin binding assay

Bacteria were cultured on blood agar plates with 5% sheep blood (Thermo Scientific Oxoid™, Waltham, MA, USA; PB5012A) or Gram-positive selective CAP agar with 5% sheep blood (Thermo Scientific Oxoid™, Waltham, MA, USA; PB5082A). A disk of vancomycin (5 μg) was laid on the border of the agar plate to be used as control for functional vancomycin resistance. However, for the vancomycin-binding experiments, bacteria were harvested as far as possible from the disk, to avoid possible competition effects between labelled and non-labelled vancomycin.

BODIPY™-labelled vancomycin (Thermo Scientific, Waltham, MA. USA; V34850) was used to measure the affinity of vancomycin for peptidoglycan precursors in the cell wall of the bacterial isolates of interest. Labelled vancomycin was resuspended at 1 mg/mL, corresponding with 8.4 μg/μL of vancomycin. Prior to use, the BODIPY™-labelled vancomycin was diluted in Tryptic Soy Broth (TSB; Xebios Diagnostics GmbH, Düsseldorf, Germany) at 840 μg/mL final concentration, ready to be added to the bacterial suspensions. A dilution series of the same BODIPY™-labelled vancomycin in TSB broth was prepared to be used as a semi-quantitative calibration curve and to account for possible decrease in fluorescence intensity over time. *E. faecium* were harvested and resuspended at 0.5 McFarland in TSB broth, 90 μL hereof was added to 96-wells clear bottomed wells (Greiner Bio-one, Alphen aan den Rijn, Netherlands, #655090) and to 96-wells white-walled wells (Greiner Bio-one, Alphen aan den Rijn, Netherlands, #655075). Hereto, 10 μL of TSB broth was added to half of the wells, and 10 μL of TSB-labelled-vancomycin was added to the other half of the wells, culminating in a final concentration of either 0 μg/mL or 84 μg/mL vancomycin.

Absorbance (OD_600_) was measured in 96-wells clear bottomed plates with a spectrophotometer (Spectramax® iD3; Molecular Devices, San Jose, CA. USA), set at 35 °C, with gentle shaking prior to each measurement. BODIPY™ fluorescence (Ex_480_ Em_520_) was measured in white-walled plates. Binding was measured by harvesting all bacterial cells in Eppendorf tubes, briefly centrifuging the suspension at maximum speed (1 minute at >10,000g) to separate the supernatants (top 90 μL) and bacterial pellet (bottom 10 μL). Separated fractions were measured for BODIPY™ fluorescence. Binding was defined as the fraction of recovered fluorescence intensity in the cells relative to the fluorescence intensity of the suspension. The relative amount of BODIPY™ fluorescence incorporated in the cell pellet was compared between the resistant phenotype and the susceptible phenotype of each specific bacterial isolate.

### Statistics

Statistical analysis were performed using unpaired t-tests with Welch’s correction, except for the ΔCt analysis, which was analysed using two-way ANOVA. Analyses were conducted in GraphPad Prism (v10.4.0).

### Ethics statement

Clinical isolates were obtained as part of routine microbiology diagnostics and processed anonymously. No informed consent was required. The study was approved by the local research committee of Rijnstate (#2021–1949) and the medical ethics committee of Maastricht UMC+ (2022–3239).

## Results

We screened enterococcal isolates from both long-term storage collections and routine clinical cultures. The Maastricht UMC+ collection primarily comprised phenotypically vancomycin-susceptible isolates, whereas the Rijnstate collection included all enterococci cultured during the study period as well as all isolates archived in long-term storage. The latter collection predominantly consisted of isolates from presumed sterile sites, such as prosthetic joint infections.

In total, 477 isolates were analyzed: 250 from Rijnstate and 228 from Maastricht UMC + . The species distribution included 234 *E. faecalis*, 227 *E. faecium*, 4 *E. gallinarum*, 2 *E. casseliflavus*, 3 *E. avium*, 3 *E. raffinosum*, 2 *E. durans*, 1 *E. hirae* and 1 undetermined *Enterococcus* species.

Vancomycine resistance genes were detected in 26 of 477 isolates (5.5%) (Table 1). These comprised 8/234 *E. faecalis* (3.4%; *vanA*-positive), 13/227 *E. faecium* (5.7%; all *vanB*-positive), 3/4 *E. gallinarum* (75%; *vanC1*-positive), 2/2 *E. casseliflavus* (100%; *vanC2/3*-positive). Among these 26 isolates, 20 (76.9%) displayed phenotypic vancomycin resistance: 8/8 *vanA*-positive *E. faecalis* (100%), 9/13 *vanB*-positive *E. faecium* (69.2%), 2/3 *vanC1*-positive *E. gallinarum* (66.7%), and 1/2 vanC2/3-positive *E. casseliflavus* (50%). The remaining 6 isolates (23.1%) carried *van* genes but remained phenotypically susceptible to vancomycin. These included 4 *E. faecium* isolates with *vanB*, 1 *E. gallinarum* with *vanC1* and 1 *E. casseliflavus* with *vanC2/3* (Table 1).

**Table 1:** Overview of molecular and phenotypic results in enterococci.

Species	Site of origin									Van resistance gene (PCR)						Vancomycin susceptibility if PCR-positive **	
	CSF	BAL	Pus/ Punction	Fluid (GI)	Urine	Rectum	Tissue	Blood	Unknown	Neg	*vanA*	*vanB*	*vanC1*	*vanC2/3*	*vanD*	S	R
*E. faecalis*	7	0	2	1	7	5	20	191	1	226	8	0	0	0	0	0	8
*E. faecium*	2	4	1	3	0	14	3	200	0	214	0	13	0	0	0	4	9
*E. gallinarum*	0	0	0	0	0	0	0	4	0	1	0	0	3	0	0	1	2
*E. casseliflavus*	0	0	0	0	0	0	1	1	0	0	0	0	0	2	0	1	1
*E. avium*	0	0	0	0	0	0	1	2	0	3	0	0	0	0	0	0	0
*E. raffinosum*	0	0	0	0	0	0	2	1	0	3	0	0	0	0	0	0	0
*E. durans*	0	0	0	0	0	0	0	2	0	2	0	0	0	0	0	0	0
*E. hirae*	0	0	0	0	0	0	0	1	0	1	0	0	0	0	0	0	0
Enterococcus spp*	0	0	0	0	0	0	0	1	0	1	0	0	0	0	0	0	0

All fields that are positive (i.e., greater than 0) are marked in grey for visual purposes only. CSF: Cerebrospinal fluid; BAL: Bronchoalveolar lavage fluid; Fluid (GI): peritoneal fluids; vanco-S/-R: vancomycin susceptible (MIC ≤ 4 mg/L) or resistant (>4mg/L). * Enterococcus species, not further determined by MALDI-ToF. ** The vancomycin phenotype of the PCR positive isolates was looked up in the laboratory information system. The vancomycin gene PCR was performed retrospectively and thus not reported to the requesting physician.

### Induction of resistance in VVE

**Fig 1:**
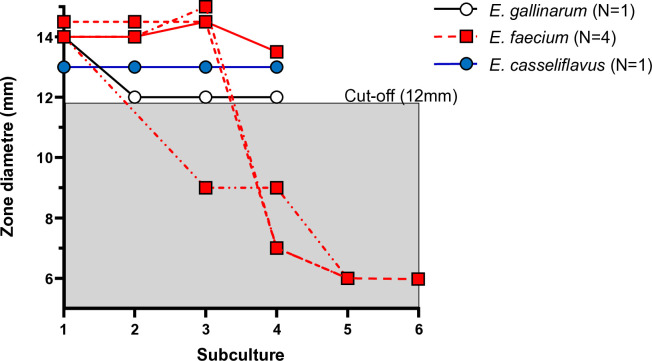
Induction of resistance in *van* gene containing enterococci.

Six putative VVE isolates – defined as isolates positive for *vanA/B/C/D* genes by PCR but phenotypically susceptible to vancomycin – were available for induction experiments: four *E. faecium*, one *E. gallinarum* and one *E. casseliflavus*. Three *E. faecium* isolates developed stable vancomycin resistance during the induction process, with inhibition zones around a 5 μg vancomycin disk decreasing from 14–15 mm to ≤6 mm, with growth extending to the disk margin (Fig 1, S2 Table). These three isolates thus fulfilled the criteria for vancomycin-variable enterococci. Moreover, the three isolates were derived from the same laboratory, and cultured from a rectal swab taken as part of screening procedure for VRE carriage in patients (S2 Table). All isolates remained susceptible to gentamicin and teicoplanin (S2 Table). However, when vancomycin and teicoplanin disks were placed in close proximity (12–16 mm apart), teicoplanin resistance was induced by vancomycin exposure (S2 Table). The retained baseline susceptibility to teicoplanin indicates that vanB was not constitutively expressed in the resistant phenotype.

### Genomics

Whole genome sequencing was performed on three *vanB*-positive *E. faecium* isolates with a vancomycin-susceptible phenotype (passage 1) and their corresponding induced vancomycin-resistant derivatives (passage 6). Combined short- and long-read sequencing (S4 Table) showed that all three isolates belonged to sequence type ST117 CC17. Each isolate carried a single *vanB2* operon on a 3 Mbp contig, consistent with chromosomal integration.

In addition to *vanB2*, all isolates encoded multiple resistance determinants, including aac [3]-ENT, eat(A), msr(C), erm(b), and dfrG. Several virulence factors were also detected, notably *acm* (collagen adhesin), *ecbA* (collagen binding), *fss3* (fibrinogen binding) and *sgrA* (cell wall anchoring protein). SNP-based analysis clustered the three isolates within a single clonal group (8–11 SNP differences; cut-off 20 SNP; see also S3 Table).

Pairwise comparison of the resistant derivatives with their susceptible progenitors revealed three unique mutations, one in each resistant isolate (S3 Table, S1 Fig). These mutations were novel and located in genes encoding the VanR and VanS regulatory proteins of the *vanB* operon.

#### RNA expression assay.

We hypothesized that the mutations in vanR and VanS may lead to enhanced or constitutive *vanB* expression. To test this, *vanB* RNA levels were compared with the housekeeping gene *recG* under conditions with and without vancomycin stimulation (S5A Table).

For each isolate, bacteria harvested close to the vancomycin disk (‘stimulated’) were compared with those harvested from the opposite edge of the blood agar plate (‘non-stimulated’), ensuring similar growth conditions apart from vancomycin exposure.

**Fig 2:**
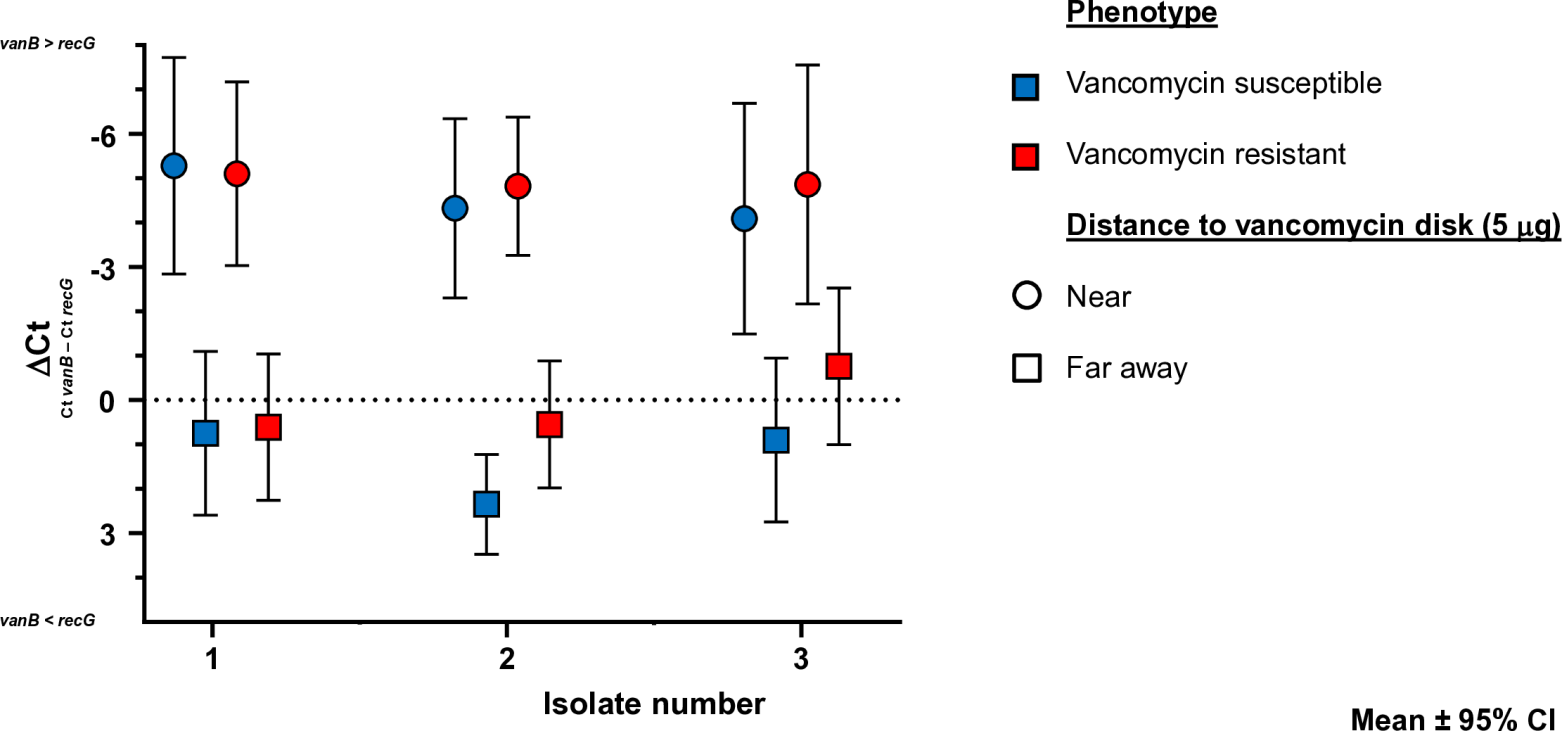
Relative *vanB* RNA expression per isolate per condition. Shown are the relative expression levels of *vanB* RNA relative to *recG* RNA for three *Enterococcus faecium* isolates, each in its vancomycin susceptible phenotype (red colors) and its vancomycin resistant phenotype (blue colors), when these isolates were cultured near (circles) or far away (squares) from a vancomycin disk (5 μg). The y-axis shows the difference in Ct values between the *vanB* PCR and the *recG* PCR. ΔCt values <0 indicate higher responsiveness of *vanB* expression while ΔCt values >0 indicate stronger response of *recG* than *vanB*. Results are plotted as mean ± 95% confidence interval.

Semi-quantitative PCR demonstrated that, at the RNA level, vancomycin stimulation induced a marked upregulation of *vanB* expression independent of phenotypic resistance (Fig 2, S5A Table). No differences were seen in Ct values of *vanB* and *recG* in the total nucleic acids used for the experiments (S5B Table). No statistically significant differences were seen between the three isolates if grown under the same conditions and phenotype (p = 0.8126).

### Vancomycin binding assay

To confirm the functional activity of vanB in the three VVE isolates that acquired a vancomycin-resistant phenotype, we performed a vancomycin-binding assay using fluorescent BODIPY™-labeled vancomycin.

**Fig 3:**
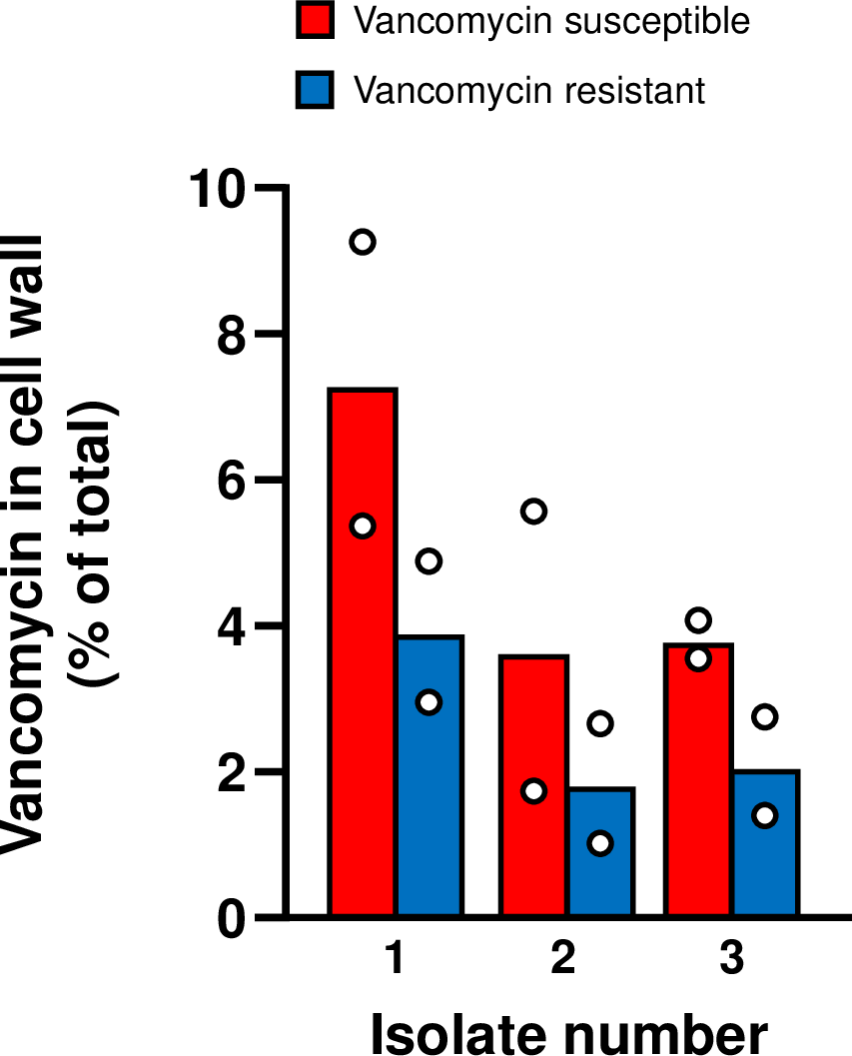
Vancomycin binding in phenotypically resistant (blue) and susceptible (red) *E. faecium* isolates ((N = 2). Different levels of vancomycin-associated fluorescence is seen between the isolates and phenotypes. Resistant isolates bound half the number of BODIPY™-vancomycin molecules relative to their vancomycin susceptible counterpart (52.8% ± 2.1%).

At 3h post-inoculation, cells reached logarithmic growth phase as determined by OD_600_ absorbance (S2A Fig), at which point fluorescence in the cell pellet was quantified as a measure of vancomycin incorporation into the cell wall using a calibration curve (S2B Fig). BODIPY™-vancomycin binding was observed in all isolates, irrespective of phenotype (Fig 3). The level of BODIPY™-vancomycin binding varied per strain. However, when directly comparing the susceptible phenotype and resistant phenotypes of each isolate, fluorescence was consistently lower in the resistant phenotype. On average, resistant phenotypes incorporated 52.8% ± 2.1% less labelled vancomycin into the cell wall compared with their susceptible counterparts (p ≤ 0.05).

## Discussion

Vancomycin-variable enterococci (VVE) represent a potential threat to infected patients, particularly if not recognized as precursors of vancomycin-resistant enterococci (VRE). In this study, we identified *vanB2*-positive, vancomycin-susceptible *E. faecium* ST117 isolates that acquired resistance under antibiotic pressure *in-vitro*. This finding highlights a third route by which patients may acquire VRE colonization: i) ingestion and colonization by VRE, ii) *in vivo* acquisition of resistance in *Enterococcus* spp., and iii) progression of VVE to VRE [8,14,24].

Most of the previously described VVE are *vanA*-positive strains with defective vanR/vanS regulatory systems [11,13,15,16,25–27]. Resistance in these isolates is often driven by constitutive or enhanced *vanA* expression, through promotor deletions, increased plasmid copy number, or impaired deactivation of vanR/vanS signaling [9,24,26,28,29]. In contrast, the *vanB*-positive *E. faecium* isolates described here developed resistance through three distinct, novel mutations: two in vanS and one in vanR. Each mutation alone was sufficient to confer vancomycin resistance by reducing drug binding to the cell wall. Importantly, this phenotype was not linked to elevated *vanB* RNA expression, and the isolates remained susceptible to teicoplanin, excluding constitutive vanB activity as a mechanism.

Genomic analysis and metadata indicated that the VVE isolates likely belonged to the same outbreak. The overall prevalence of VVE among phenotypically susceptible *E. faecium* was low (1.8%; 4/227), but three of four isolates (75%) converted to a resistant phenotype within a few passages under vancomycin pressure. Because these isolates were clonally related, the prevalence estimate must be interpreted with caution. Nonetheless, our findings provide clear evidence that VVE exist in Dutch clinical settings and can escape detection when laboratories rely solely on phenotypical assays. This limitation is clinically relevant, as both VRE and VVE may be missed by selective agars or culture-based susceptibility testing due to assay- or strain-specific factors [15,30,31].

Experiences from Denmark illustrate the potential impact: the prevalence of a specific VVE clone (ST1421-CT1134) rose from 3% in 2017, to 34% in 2018, and 44% by early 2019, largely due to diagnostic algorithms that failed to detect these isolates, ultimately prompting a national policy shift toward molecular screening for *vanA* [16]. Our findings suggest that such screening policies should not be restricted to *vanA* but should also include *vanB* [15]. Laboratories may use in-house molecular assays targeting multiple *van* genes [8] or commercial platforms such as the Cepheid GeneXpert, which detects *vanA* and *vanB* [32], although use in this context may be off-label.

Taken together, our results demonstrate that vancomycin-susceptible VVE can evolve into clinically relevant VRE under antibiotic selection pressure *in vitro*. Reports of *in vivo* emergence remain rare [14], but the possibility cannot be excluded. Considering the low prevalence but potentially severe consequences, we recommend targeted molecular screening in cases where the same *Enterococcus* species is repeatedly cultured from sterile or deep wound during ongoing vancomycin therapy. Under current epidemiological conditions, universal screening of all enterococci for vancomycin-resistance genes is neither cost-effective nor justified. However, selective molecular testing can enhance diagnostic accuracy, guide culture conditions, and prevent VVE from escaping clinical detection [16,30].

## Supporting information

S1 Table Primer/probe sequences used in this study. (DOCX)

S2 Table Overview of antibiotic resistance results for the enterococci tested in this study. (DOCX)

S3 Table Overview of SNP difference between the three vancomycin variable enterococci studied, both in original vancomycin-susceptible phenotype and in vancomycin-resistant phenotype. (DOCX)

S4 Table Statistics of WGS assemblies of the three vancomycin variable enterococci. (DOCX)

S5 Table Raw data pertaining to RNA transcripts. (DOCX)

S1 Fig Graphical depiction of the *vanB2* operon in this study. The *vanB2* operon found in the vancomycin-variable enterococci in this study is depicted together with the mutations identified in the *vanS* and *vanR* genes.
(TIF)

S2A Fig Growth curve of isolates of interest as determined by absorbance (OD_600_). Bacterial suspensions were incubated at 35 °C and growth was followed by measuring the absorbance (OD_600_) with a spectrophotometer, with gentle shaking prior to each measurement. Each bacterial isolate and condition was plated in duplicate.
(TIF)

S2B Fig Calibration curve of BODIPY™-labelled vancomycin. A dilution series of BODIPY™-labelled vancomycin was prepared in tryptic soy broth, and measured at three time points: 0h, 1h and 2h.
(TIF)
