# Supplementary material for: Vancomycin variable Enterococci in the Netherlands (2018–2023) and the mechanism of resistance induction
Source: PLoS One. 2026 Feb 6;21(2):e0342092. doi: 10.1371/journal.pone.0342092 (PMC12880688; doi:10.1371/journal.pone.0342092)
Supplement: S1 Table — (DOCX) [file pone.0342092.s001.docx]

**Supplemental materials**

S1 Table: Primer/probe sequences used in this study

| Name | Sequence | Fluorphore | Quencher | Final concentration | μL* | PCR efficiency  (slope ±SD) | Reference |
| --- | --- | --- | --- | --- | --- | --- | --- |
| recG-fw (RS) | GCA ACT AAG ATA CAG TAG GAA GCA TCA |  |  | 300nM | 0.9 | 94.3%  (-3.38±0.40) | (1) |
| recG-rev1 (RS) | TGC GGA TCG ATT TGG TTT G |  |  | 300nM | 0.9 |  |  |
| recG-rev2 (RS) | TGC GGA TCG ATT TGG ATT G |  |  | 300nM | 0.9 |  |  |
| recG-FAM (RS) | CGC GTC CAA CAC GTC CAC GAA | 6FAM | BHQ1 | 200nM | 0.4 |  |  |
|  |  |  |  |  |  |  |  |
| vanA-fw (RS) | CCC GGT TTC ACG TCA TAC AGT |  |  | 400nM | 0.12 | 113%  (-3.20±0.19) | (1) |
| vanA-rev (RS) | CAA TCA GTT CGG GAA GTG CAA |  |  | 400nM | 0.12 |  |  |
| vanA-VIC (RS) | CCT GCA GCG GCC ATC ATA CGG | VIC replacement | BHQ1 | 200nM | 0.12 |  |  |
|  |  |  |  |  |  |  |  |
| vanB-fw1 (RS) | CAA TAC AAA CAG ACC CTG TAT CG |  |  | 300nM | 0.09 | 86%  (-3.50±0.29) | (1) |
| vanB-fw2 (RS) | CAA TAC AAA CAG CCC CTG TAT CG |  |  | 300nM | 0.09 |  |  |
| vanB-rev1 (RS) | CGG CGT ATT GAC GTG GCT TT |  |  | 300nM | 0.09 |  |  |
| vanB-rev2 (RS) | CGG CGT ATT GAT GTG GCT TT |  |  | 300nM | 0.09 |  |  |
| vanB-NED (RS) | TCC TCC CCG CAT TTG CCA TGC | NED replacement | BHQ2 | 200nM | 0.12 |  |  |
|  |  |  |  |  |  |  |  |
| vanA-fw (MUMC+) | GCCGGAAAAAGGCTCTGAA |  |  | 800nM |  | 102.4%  -3.30 | (2) |
| vanA-rev (MUMC+) | TCCTCGCTCCTCTGCTGAA |  |  | 800nM |  |  |  |
| vanA-FAM (MUMC+) | ACGCAGTTATAACCGTTCCCGCAGACC | 6FAM | BHQ1 | 200nM |  |  |  |
|  |  |  |  |  |  |  |  |
| vanB-fw (MUMC+) | CGCAGCTTGCATGGACAA |  |  | 800nM |  | 74%  (-3.67) | (2) |
| vanB-rev (MUMC+) | GGCGATGCCCGCATT |  |  | 800nM |  |  |  |
| vanB-VIC (MUMC+) | TCACTGGCCTACATTC | VIC | MGB-NFQ | 200nM |  |  |  |
|  |  |  |  |  |  |  |  |
| vanC1-fw | CTTATGTTGGTTGCCATGTCG |  |  | 300nM |  | 74%  (-3.67±0.24) | (2) |
| vanC1-rev | CGATTGTGGCAGGATCGTT |  |  | 300nM |  |  |  |
| vanC1-NED | TGGCTCTTGCATCAACTTGCTGATACCA | NED | BHQ2 | 200nM |  |  |  |
|  |  |  |  |  |  |  |  |
| vanC2/3-fw | GCACTCCAATCATCTCCCTATG |  |  | 300nM |  | 75%  (-3.67±0.27) | (2) |
| vanC2/3-rev | CAYGTGTCTTGTCGGATGTT |  |  | 300nM |  |  |  |
| vanC2/3-JOE | TAYGACCTCTCTTTGATCGGGATCRCC | JOE | BHQ1 | 200nM |  |  |  |
|  |  |  |  |  |  |  |  |
| vanD-fw | GCCATACTGGGAAAYGRAAA |  |  | 300nM |  | 69%  (-3.79±0.29) | (2) |
| vanD-rev | CAGCCAAGTAYCCGGTAAATC |  |  | 300nM |  |  |  |
| vanD-FAM | TCCGGCTGTGCTTCCTGATGRATC | 6FAM | BHQ1 | 200nM |  |  |  |
|  |  |  |  |  |  |  |  |

* Volume per PCR mix using the standard concentrations for Primers (100μM) and probes (15μM). PCR efficiency was calculated based on the slope of a dilution series.

**References**

1. van der Zee A, Ossewaarde JM. 'search and control'-strategie voor inperking van bijzonder resistente micro-organismen. Ned Tijdschr Med Microbiol. 2016;24(4):164 - 9.

2. Flipse J, von Wintersdorff CJH, van Niekerk JM, Jamin C, van Tiel FH, Hasman H, et al. Appearance of vanD-positive Enterococcus faecium in a tertiary hospital in the Netherlands: prevalence of vanC and vanD in hospitalized patients. Sci Rep. 2019;9(1):6949.
