# Supplementary material for: Vancomycin variable Enterococci in the Netherlands (2018–2023) and the mechanism of resistance induction
Source: PLoS One. 2026 Feb 6;21(2):e0342092. doi: 10.1371/journal.pone.0342092 (PMC12880688; doi:10.1371/journal.pone.0342092)
Supplement: S2 Table — (DOCX) [file pone.0342092.s002.docx]

S2 Table: overview of antibiotic resistance results for the Enterococci tested in this study.

| Species | Clinical specimen | van gene PCR  (vanA/vanB/vanC/vanD) | Vancomycin | | | Amoxicillin | | | Number of isolates | Vancomycin resistance induction. Diameter of inhibition zone around 5 μg disk (mm), per repetition | | | | | | Inhibition zone around 30μg disk gentamycin (mm), per repetition | | Inhibition zone diameter around 30μg disk teicoplanin (mm), per repetition | | vancomycin-induced resistance against teicoplanin? | |
| --- | --- | --- | --- | --- | --- | --- | --- | --- | --- | --- | --- | --- | --- | --- | --- | --- | --- | --- | --- | --- | --- |
|  |  |  | Vitek | Test strip* | Disk (mm) | Vitek | Test strip* | Disk (mm) |  | 1st | 2nd | 3rd | 4th | 5th | 6th | 1st | 6th | 1st | 6th | 1st | 6th |
| *E. casseliflavus* | Tissue | vanC2/3 | 2.0 |  |  | ≤2 |  |  | 1 | 13 | 13 | 13 | 13 |  |  |  |  |  |  |  |  |
| *E. casseliflavus* | Blood | vanC2/3 | 4.0 |  |  | ≤2 |  |  | 1 | 11.5 |  |  |  |  |  |  |  |  |  |  |  |
| *E. faecalis* | Rectum | vanA |  |  | 6 |  |  | 11 | 1 |  |  |  |  |  |  |  |  |  |  |  |  |
| *E. faecalis* | Rectum | vanA | ≥32 |  |  | ≤2 |  |  | 1 |  |  |  |  |  |  |  |  |  |  |  |  |
| *E. faecalis* | Rectum | vanA | ≥32 |  |  | ≥32 |  |  | 1 |  |  |  |  |  |  |  |  |  |  |  |  |
| *E. faecalis* | Urine | vanA | ≥32 |  |  | ≤2 |  |  | 2 |  |  |  |  |  |  |  |  |  |  |  |  |
| *E. faecalis* | Urine | vanA | ≥32 |  | 6 | ≤2 |  |  | 3 |  |  |  |  |  |  |  |  |  |  |  |  |
| *E. faecium* | Rectum | vanB |  |  | 14.5 | ≥32 |  |  | 1 | 14.5 | 14.5 | 14.5 | 7 | 6 | 6 | 19 | 17 | 19 | 20 | Yes | Yes |
| *E. faecium* | Rectum | vanB |  |  | 14 | ≥32 |  |  | 1 | 14 | 14 | 15 | 7 | 6 | 6 | 17 | 18 | 20 | 20 | Yes | Yes |
| *E. faecium* | Rectum | vanB |  |  | 14 | ≥32 |  |  | 1 | 14 |  | 9 | 9 | 6 | 6 | 19 | 17 | 18 | 19 | Yes | Yes |
| *E. faecium* | Pus / Punction | vanB | ≤0.5 | 0.38 | 16 | ≥32 |  |  | 1 | 14 | 14 | 14.5 | 13.5 |  |  |  |  |  |  |  |  |
| *E. faecium* | Rectum | vanB | 1.0 | 4.0 |  | ≥32 |  |  | 1 | 11 |  |  |  |  |  |  |  |  |  |  |  |
| *E. gallinarum* | Blood | vanC1 | 2.0 |  |  | ≤2 |  |  | 1 | 14 | 12 | 12 | 12 |  |  |  |  |  |  |  |  |
| *E. gallinarum* | Blood | vanC1 | 8.0 |  |  | ≤2 |  |  | 2 |  |  |  |  |  |  |  |  |  |  |  |  |
|  |  |  |  |  |  |  |  |  |  |  |  |  |  |  |  |  |  |  |  |  |  |
| *Enterococ species* | Blood | negative | ≤0.5 |  |  | 16 |  |  | 1 |  |  |  |  |  |  |  |  |  |  |  |  |
| *E. raffinosum* | Blood | negative | ≤0.5 |  |  | 8 | 8.0 |  | 1 |  |  |  |  |  |  |  |  |  |  |  |  |
| *E. raffinosum* | Tissue | negative |  |  | 17 |  |  |  | 1 |  |  |  |  |  |  |  |  |  |  |  |  |
| *E. raffinosum* | Tissue | negative |  |  | 20 |  |  |  | 1 |  |  |  |  |  |  |  |  |  |  |  |  |
| *E. avium* | Blood | negative | ≤0.5 |  |  | ≤2 |  |  | 2 |  |  |  |  |  |  |  |  |  |  |  |  |
| *E. avium* | Tissue | negative |  |  | 20 |  |  |  | 1 |  |  |  |  |  |  |  |  |  |  |  |  |
| *E. durans* | Blood | negative | ≤0.5 |  |  | ≤2 |  |  | 2 |  |  |  |  |  |  |  |  |  |  |  |  |
| *E. faecalis* | Rectum | negative | 1.0 |  |  |  |  |  | 1 |  |  |  |  |  |  |  |  |  |  |  |  |
| *E. faecalis* | Blood | negative | ≤0.5 |  |  | ≤2 |  |  | 7 |  |  |  |  |  |  |  |  |  |  |  |  |
| *E. faecalis* | Blood | negative | ≤0.5 |  |  | ≥32 |  |  | 2 |  |  |  |  |  |  |  |  |  |  |  |  |
| *E. faecalis* | Blood | negative | 1.0 |  |  | ≤2 |  |  | 126 |  |  |  |  |  |  |  |  |  |  |  |  |
| *E. faecalis* | Blood | negative | 1.0 | 2.0 |  | ≤2 |  |  | 1 |  |  |  |  |  |  |  |  |  |  |  |  |
| *E. faecalis* | Blood | negative | 1.0 |  | 13 | ≤2 |  | 12 | 1 |  |  |  |  |  |  |  |  |  |  |  |  |
| *E. faecalis* | Blood | negative | 1.0 |  | 15 | ≤2 |  |  | 2 |  |  |  |  |  |  |  |  |  |  |  |  |
| *E. faecalis* | Blood | negative | 1.0 |  | 16 | ≤2 |  | 18 | 2 |  |  |  |  |  |  |  |  |  |  |  |  |
| *E. faecalis* | Blood | negative | 2.0 |  |  | ≤2 |  |  | 43 |  |  |  |  |  |  |  |  |  |  |  |  |
| *E. faecalis* | Blood | negative | 2.0 |  |  | ≤2 | 1.5 |  | 1 |  |  |  |  |  |  |  |  |  |  |  |  |
| *E. faecalis* | Blood | negative | 2.0 | 2.0 | 13 | ≤2 |  |  | 1 |  |  |  |  |  |  |  |  |  |  |  |  |
| *E. faecalis* | Blood | negative |  |  | 21 |  |  |  | 1 |  |  |  |  |  |  |  |  |  |  |  |  |
| *E. faecalis* | CSF | negative | 1.0 |  |  | ≤2 |  |  | 5 |  |  |  |  |  |  |  |  |  |  |  |  |
| *E. faecalis* | CSF | negative | 2.0 |  |  | ≤2 |  |  | 2 |  |  |  |  |  |  |  |  |  |  |  |  |
| *E. faecalis* | Urine | negative | 2.0 |  |  | ≤2 |  |  | 1 |  |  |  |  |  |  |  |  |  |  |  |  |
| *E. faecalis* | Urine | negative | ≥8 | 16.0 | 14 | ≤2 |  |  | 1 |  |  |  |  |  |  |  |  |  |  |  |  |
| *E. faecalis* | Pus / Punction | negative | 2.0 |  |  | ≤2 |  |  | 1 |  | |  |  |  |  |  |  |  |  |  |  |
| *E. faecalis* | Fluid (GI) | negative | 1.0 |  |  | ≤2 |  |  | 1 |  |  |  |  |  |  |  |  |  |  |  |  |
| *E. faecalis* | Tissue | negative | 1.0 |  |  | ≤2 |  |  | 13 |  |  |  |  |  |  |  |  |  |  |  |  |
| *E. faecalis* | Tissue | negative |  |  | 13 | ≤2 |  |  | 1 |  |  |  |  |  |  |  |  |  |  |  |  |
| *E. faecalis* | Tissue | negative | 2.0 |  |  | ≤2 |  |  | 6 |  |  |  |  |  |  |  |  |  |  |  |  |
| *E. faecium* | BAL | negative | ≤0.5 |  | 17 | ≥32 |  |  | 1 |  |  |  |  |  |  |  |  |  |  |  |  |
| *E. faecium* | BAL | negative | ≤0.5 |  | 18 | ≥32 |  |  | 1 |  |  |  |  |  |  |  |  |  |  |  |  |
| *E. faecium* | BAL | negative | ≤0.5 |  | 19 | ≥32 |  |  | 2 |  |  |  |  |  |  |  |  |  |  |  |  |
| *E. faecium* | Blood | negative | ≤0.5 |  |  | ≤2 |  |  | 2 |  |  |  |  |  |  |  |  |  |  |  |  |
| *E. faecium* | Blood | negative | ≤0.5 |  | 17 | ≤2 |  |  | 1 |  |  |  |  |  |  |  |  |  |  |  |  |
| *E. faecium* | Blood | negative | ≤0.5 |  | 18 | ≤2 |  |  | 2 |  |  |  |  |  |  |  |  |  |  |  |  |
| *E. faecium* | Blood | negative | ≤0.5 |  | 19 | ≤2 |  |  | 1 |  |  |  |  |  |  |  |  |  |  |  |  |
| *E. faecium* | Blood | negative | ≤0.5 |  | 18 | 8 |  |  | 1 |  |  |  |  |  |  |  |  |  |  |  |  |
| *E. faecium* | Blood | negative | ≤0.5 |  | 18 | 8 | >256 |  | 1 |  |  |  |  |  |  |  |  |  |  |  |  |
| *E. faecium* | Blood | negative | ≤0.5 |  |  | 16 |  |  | 1 |  |  |  |  |  |  |  |  |  |  |  |  |
| *E. faecium* | Blood | negative | ≤0.5 |  | 18 | 16 |  |  | 1 |  |  |  |  |  |  |  |  |  |  |  |  |
| *E. faecium* | Blood | negative | ≤0.5 |  |  | ≥32 |  |  | 60 |  |  |  |  |  |  |  |  |  |  |  |  |
| *E. faecium* | Blood | negative | ≤0.5 |  | 13 | ≥32 |  |  | 1 |  |  |  |  |  |  |  |  |  |  |  |  |
| *E. faecium* | Blood | negative | ≤0.5 |  | 14 | ≥32 |  |  | 1 |  |  |  |  |  |  |  |  |  |  |  |  |
| *E. faecium* | Blood | negative | ≤0.5 |  | 15 | ≥32 |  |  | 7 |  |  |  |  |  |  |  |  |  |  |  |  |
| *E. faecium* | Blood | negative | ≤0.5 |  | 16 | ≥32 |  |  | 18 |  |  |  |  |  |  |  |  |  |  |  |  |
| *E. faecium* | Blood | negative | ≤0.5 |  | 17 | ≥32 |  |  | 32 |  |  |  |  |  |  |  |  |  |  |  |  |
| *E. faecium* | Blood | negative | ≤0.5 |  | 18 | ≥32 |  |  | 41 |  |  |  |  |  |  |  |  |  |  |  |  |
| *E. faecium* | Blood | negative | ≤0.5 |  | 19 | ≥32 |  |  | 13 |  |  |  |  |  |  |  |  |  |  |  |  |
| *E. faecium* | Blood | negative | ≤0.5 |  | 20 | ≥32 |  |  | 8 |  |  |  |  |  |  |  |  |  |  |  |  |
| *E. faecium* | Blood | negative | 1.0 |  |  | ≤2 |  |  | 1 |  |  |  |  |  |  |  |  |  |  |  |  |
| *E. faecium* | Blood | negative | 1.0 |  | 17 | ≤2 |  |  | 1 |  |  |  |  |  |  |  |  |  |  |  |  |
| *E. faecium* | Blood | negative | 1.0 |  | 16 | ≥32 |  |  | 3 |  |  |  |  |  |  |  |  |  |  |  |  |
| *E. faecium* | Blood | negative | 1.0 |  | 18 | ≥32 |  |  | 1 |  |  |  |  |  |  |  |  |  |  |  |  |
| *E. faecium* | Blood | negative | 2.0 |  | 13 | ≥32 |  |  | 1 |  |  |  |  |  |  |  |  |  |  |  |  |
| *E. faecium* | Blood | negative | ≥32 |  | 19 | ≥32 |  |  | 1 |  |  |  |  |  |  |  |  |  |  |  |  |
| *E. faecium* | CSF | negative | ≤0.5 |  | 18 | ≥32 |  |  | 1 |  |  |  |  |  |  |  |  |  |  |  |  |
| *E. faecium* | CSF | negative | ≤0.5 |  | 19 | ≥32 |  |  | 1 |  |  |  |  |  |  |  |  |  |  |  |  |
| *E. faecium* | Fluid (GI) | negative | ≤0.5 |  |  | ≥32 |  |  | 2 |  |  |  |  |  |  |  |  |  |  |  |  |
| *E. faecium* | Pus / Punction | negative | ≤0.5 |  |  | ≥32 |  |  | 1 |  |  |  |  |  |  |  |  |  |  |  |  |
| *E. faecium* | Tissue | negative | ≤0.5 |  |  | ≥32 |  |  | 3 |  |  |  |  |  |  |  |  |  |  |  |  |
| *E. gallinarum* | Blood | negative | 4.0 |  |  | ≤2 |  |  | 1 |  |  |  |  |  |  |  |  |  |  |  |  |
| *E. hirae* | Blood | negative | ≤0.5 |  |  | ≤2 |  |  | 1 |  |  |  |  |  |  |  |  |  |  |  |  |
| *Data no longer available in the clinical laboratory information system due to various reasons* | | | | | | | | 19 | |  |  |  |  |  |  |  |  |  |  |  |  |

*BAL: Bronchoalveolar lavage fluid; CSF: Cerebrospinal fluid; Fluid (GI): peritoneal fluids*

**Test strip: E-Test (BioMérieux, France) or MIC Test Strip (Liofilchem, Italy).*
