# Supplementary material for: Vancomycin variable Enterococci in the Netherlands (2018–2023) and the mechanism of resistance induction
Source: PLoS One. 2026 Feb 6;21(2):e0342092. doi: 10.1371/journal.pone.0342092 (PMC12880688; doi:10.1371/journal.pone.0342092)
Supplement: S3 Table — (DOCX) [file pone.0342092.s003.docx]

S3 Table: overview of SNP difference between the three vancomycin variable enterococci studied, both in original vancomycin-susceptible phenotype and in vancomycin-resistant phenotype.

| Isolate | | 1 | 2 | 3 |  | 1 | 2 | 3 | 3  vanS, L282V (duplicate) |
| --- | --- | --- | --- | --- | --- | --- | --- | --- | --- |
|  |  | Original | Original | Original |  | vanR, T189K | vanS, G253C | vanS, L282V |  |
| 1 | Original |  | 11 | 12 |  | 11 | 9 | 10 | 9 |
| 2 | Original | 11 |  | 8 |  | 11 | 5 | 7 | 6 |
| 3 | Original | 12 | 8 |  |  | 14 | 8 | 9 |  |
| 1 | vanR, T189K | 11 | 11 | 14 |  |  | 10 | 11 | 9 |
| 2 | vanS, G253C | 9 | 5 | 8 |  | 10 |  | 7 | 6 |
| 3 | vanS, L282V | 10 | 7 | 9 |  | 11 | 7 |  | 2 |

*The table depicts the SNP difference between the three bacterial isolates in the original phenotype (vancomycin susceptible, denoted ‘original’) and the same isolate after vancomycin resistance was induced (denoted with its amino acid mutation). All combinations had <20 SNP indicating that they belong to the same cluster*
