# Supplementary material for: Vancomycin variable Enterococci in the Netherlands (2018–2023) and the mechanism of resistance induction
Source: PLoS One. 2026 Feb 6;21(2):e0342092. doi: 10.1371/journal.pone.0342092 (PMC12880688; doi:10.1371/journal.pone.0342092)
Supplement: S4 Table — (DOCX) [file pone.0342092.s004.docx]

S4 Table: Statistics of WGS assemblies of the three vancomycin variable enterococci.

| Assembly | Isolate | | #contigs ≥1000bp | Total length (≥1000bp) | #contigs | Largest contig | Total length | GC% | N_50_ | N_75_ | L_50_ | L_75_ |
| --- | --- | --- | --- | --- | --- | --- | --- | --- | --- | --- | --- | --- |
| SRR31352582 | 1 | Original | 479 | 2934795 | 566 | 37554 | 2998301 | 37,69 | 9213 | 4818 | 98 | 207 |
| SRR31352583 | 2 | Original | 247 | 3061159 | 280 | 57314 | 3084788 | 37,67 | 21855 | 12184 | 47 | 93 |
| SRR31352584 | 3 | Original | 283 | 3052813 | 315 | 67812 | 3076315 | 37,67 | 19999 | 10237 | 51 | 104 |
| SRR31352585 | 1 | vanR, T189K | 355 | 2999869 | 412 | 55819 | 3043824 | 37,67 | 14308 | 7431 | 66 | 140 |
| SRR31352586 | 2 | vanS, G253C | 195 | 3084248 | 220 | 107271 | 3102969 | 37,67 | 29096 | 15860 | 33 | 68 |
| SRR31352587 | 3 | vanS L282V | 212 | 3079066 | 239 | 89057 | 3098797 | 37,66 | 29096 | 14182 | 35 | 74 |

*The table depicts statistics of the WGS assemblies used in S3 Table.*
