# Supplementary material for: Vancomycin variable Enterococci in the Netherlands (2018–2023) and the mechanism of resistance induction
Source: PLoS One. 2026 Feb 6;21(2):e0342092. doi: 10.1371/journal.pone.0342092 (PMC12880688; doi:10.1371/journal.pone.0342092)
Supplement: S5 Table — (DOCX) [file pone.0342092.s005.docx]

S5A Table: Raw and processed data pertaining to RNA transcripts of *vanB* under conditions with high and low/absent vancomycin

| Isolate | Vancomycin phenotype and genotype | Position relative to vancomycin disk (5 μg) * | RNA (Ct ± variance) | | (ΔCt _vanB - recG_) | (ΔΔCt _near – far away_) | | Vancomycin-induced fold-change *vanB* versus *recG* |
| --- | --- | --- | --- | --- | --- | --- | --- | --- |
|  |  |  | *recG* | *vanB* |  | ΔΔCt | st dev. |  |
| 1 | Susceptible | Near | 28.21 ± 0.01 | 22.93 ± 2,34 | -5.28 | -6.03 | 1.92 | 65.3 |
|  |  | Far away | 28.56 ± 0.68 | 29.31 ± 0.66 | 0.75 |  |  |  |
| 2 | Susceptible | Near | 30.75 ± 0.18 | 26.42 ± 1,43 | -4.32 | -6.67 | 1.45 | 101.8 |
|  |  | Far away | 33.14 ± 0.27 | 35.49 ± 0.23 | 2.35 |  |  |  |
| 3 | Susceptible | Near | 29.28 ± 0.02 | 25.19 ± 2.65 | -4.09 | -4.99 | 2.00 | 31.8 |
|  |  | Far away | 31.96 ± 0.04 | 32.85 ± 1.31 | 0.90 |  |  |  |
| 1 | Resistant, (vanR, T189K) | Near | 28.32 ± 0.25 | 23.22 ± 1.44 | -5.10 | -5.71 | 1.66 | 52.3 |
|  |  | Far away | 27.74 ± 0.62 | 28.35 ± 0.45 | 0.61 |  |  |  |
| 2 | Resistant, (vanS, G253C) | Near | 29.16 ± 0.10 | 24.33 ± 0.86 | -4.82 | -5.37 | 1.33 | 41.3 |
|  |  | Far away | 32.61 ± 0.06 | 33.16 ± 0.75 | 0.55 |  |  |  |
| 3 | Resistant, (vanS, L282V) | Near | 29.51 ± 1.19 | 24.66 ± 1.67 | -4.86 | -5.62 | 2.02 | 49.2 |
|  |  | Far away | 32.11 ± 0.55 | 32.87 ± 0.68 | 0.76 |  |  |  |

Values represent Ct values after total nucleic acids were treated with DNAse. * Bacteria are plated on a standard 90 mm blood agar with a 5 μg vancomycin disk placed close to the border of the blood agar plate. For this experiment, bacteria were harvested as close as possible to the vancomycin disk (labelled “near”) and were harvested as far away as possible from the vancomycin disk (labelled “far away”).

The ΔΔCt is calculated as: (Ct*_vanB_* - Ct*_recG_*)_near_ - (Ct*_vanB_* - Ct*_recG_*)_far away_. The variance (standard deviation^2^) of a sum or subtraction is equal to the sum of all variances. Fold-change is calculated as: 2^(-ΔΔCt).

S5B Table: Raw Ct values for total nucleic acid input prior to DNAse treatment

| Isolate | Vancomycin phenotype and genotype | Position relative to vancomycin disk (5 μg) * | DNA/RNA (Ct ± variance) | |
| --- | --- | --- | --- | --- |
|  |  |  | *recG* | *vanB* |
| 1 | Susceptible | Near | 18.76 ± 0.01 | 19.01 ± 0.01 |
|  |  | Far away | 18.41 ± 0.13 | 19.01 ± 0.00 |
| 2 | Susceptible | Near | 20.56 ± 0.00 | 20.75 ± 0.00 |
|  |  | Far away | 23.89 ± 0.02 | 24.28 ± 0.01 |
| 3 | Susceptible | Near | 19.55 ± 0.39 | 19.82 ± 0.19 |
|  |  | Far away | 21.79 ± 0.01 | 22.05 ± 0.01 |
| 1 | Resistant, (vanR, T189K) | Near | 20.20 ± 0.01 | 20.04 ± 0.04 |
|  |  | Far away | 18.25 ± 0.09 | 18.89 ± 0.09 |
| 2 | Resistant, (vanS, G253C) | Near | 20.89 ± 0.00 | 21.00 ± 0.00 |
|  |  | Far away | 22.92 ± 0.18 | 23.58 ± 0.00 |
| 3 | Resistant, (vanS, L282V) | Near | 19.85 ± 0.00 | 19.96 ± 0.19 |
|  |  | Far away | 22.63 ± 0.00 | 23.10 ± 0.00 |

Values represent Ct values of total nucleic acids (DNA/RNA) of bacterial isolates.
