# Supplementary figures and images for: Vancomycin variable Enterococci in the Netherlands (2018–2023) and the mechanism of resistance induction
Source: PLoS One. 2026 Feb 6;21(2):e0342092. doi: 10.1371/journal.pone.0342092 (PMC12880688; doi:10.1371/journal.pone.0342092)

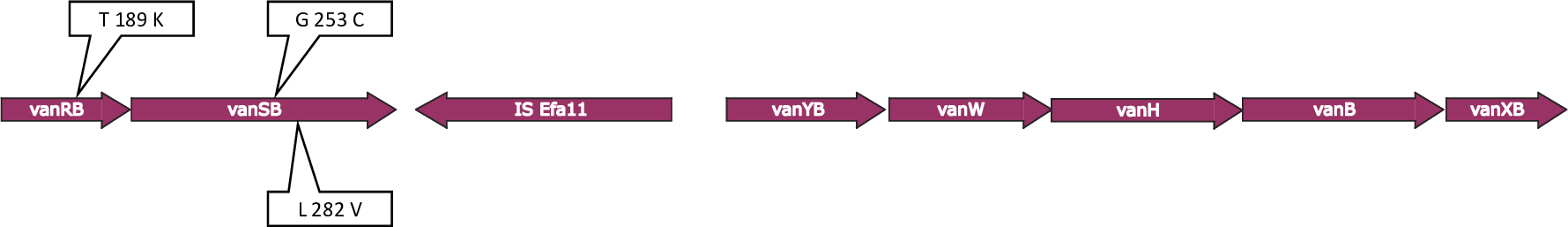

Supplement: S1 Fig — The vanB2 operon found in the vancomycin-variable enterococci in this study is depicted together with the mutations identified in the vanS and vanR genes. (TIF) [file pone.0342092.s006.tif]

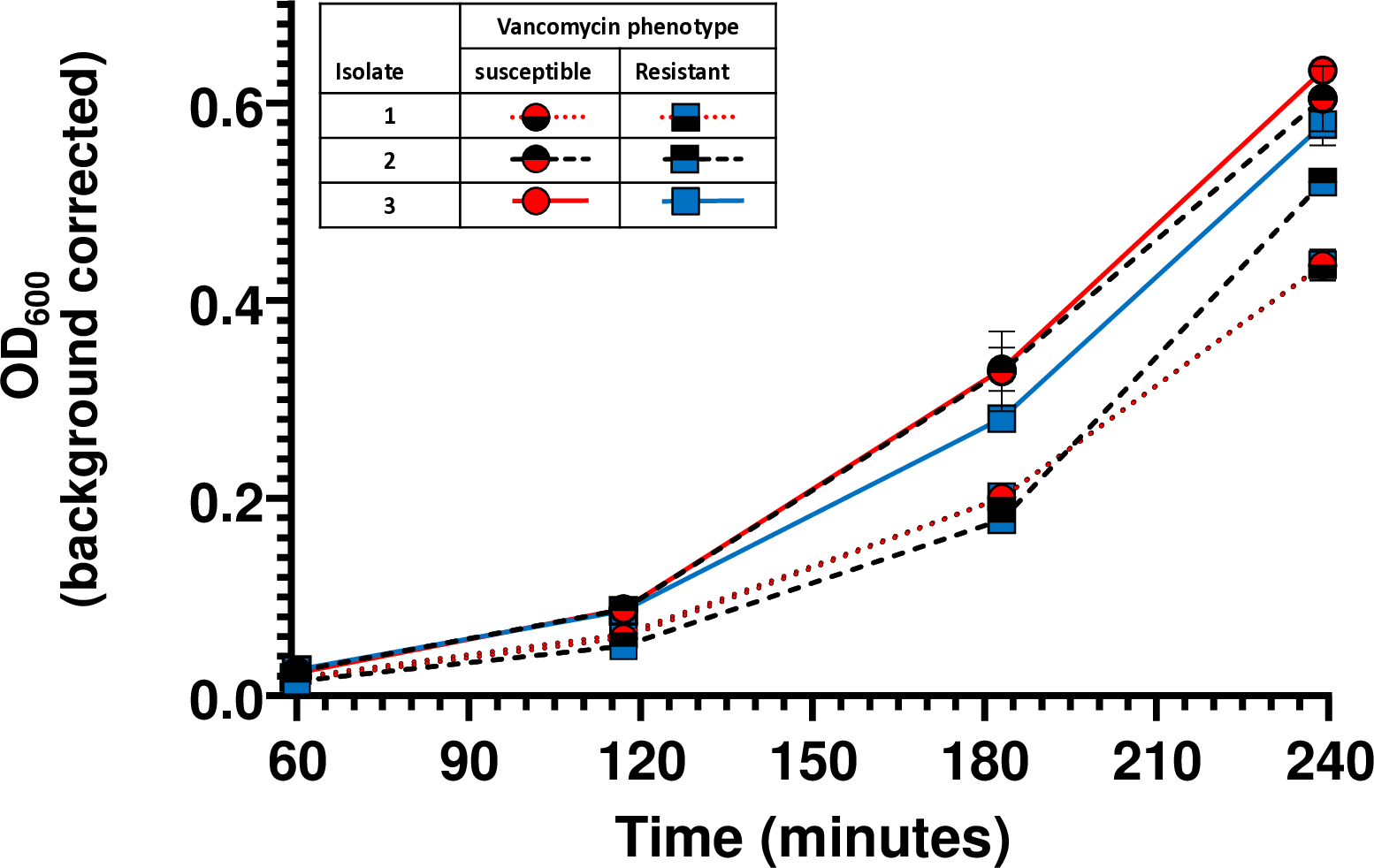

Supplement: S2A Fig — Bacterial suspensions were incubated at 35 °C and growth was followed by measuring the absorbance (OD600) with a spectrophotometer, with gentle shaking prior to each measurement. Each bacterial isolate and condition was plated in duplicate. (TIF) [file pone.0342092.s007.tif]

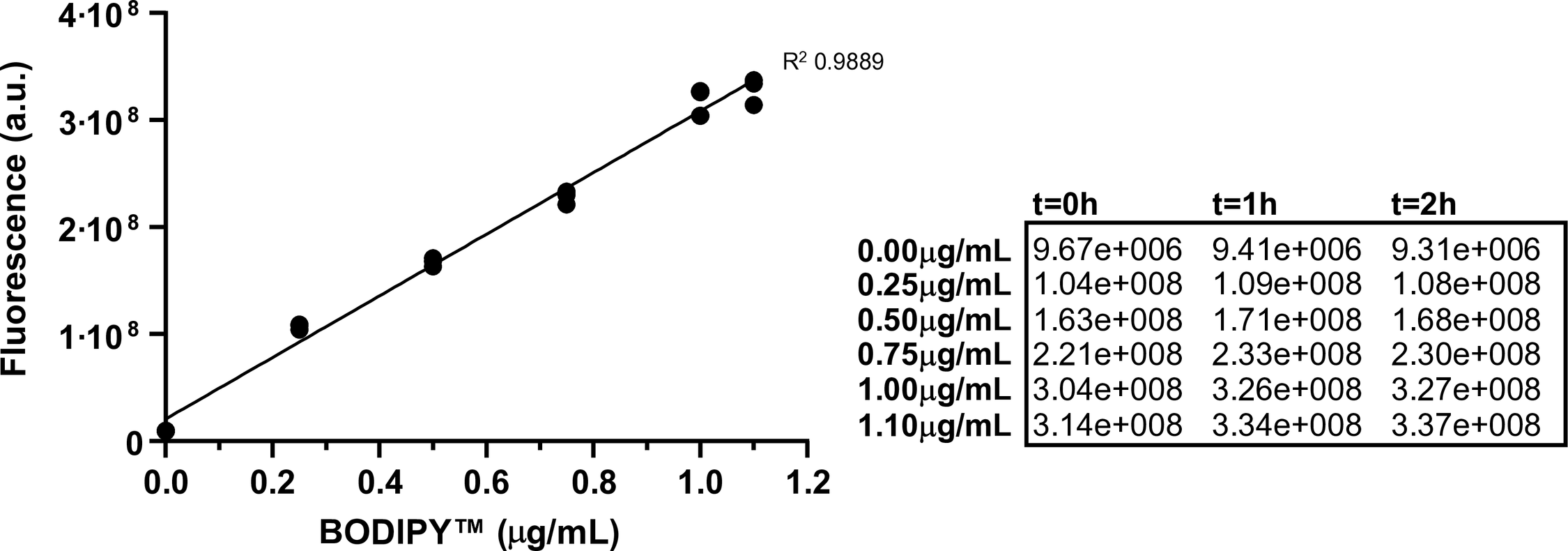

Supplement: S2B Fig — A dilution series of BODIPY™-labelled vancomycin was prepared in tryptic soy broth, and measured at three time points: 0h, 1h and 2h. (TIF) [file pone.0342092.s008.tif]
